# Special Issue Editorial: “Infections, Inflammation and Neurodegeneration in Alzheimer Disease” Infections, Neuronal Senescence, and Dementia

**DOI:** 10.3390/ijms23115865

**Published:** 2022-05-24

**Authors:** Federico Licastro

**Affiliations:** Department of Experimental, Diagnostic and Specialty Medicine (DIMES), University of Bologna, 40126 Bologna, Italy; federico.licastro@unibo.it

**Keywords:** Alzheimer’s disease, inflammation, human endogenous retrovirus activation, neuronal senescence

## Abstract

Alzheimer’s disease (AD) is a complex chronic disease of the brain characterized by several neurodegenerative mechanisms and is responsible for most dementia cases in the elderly. Declining immunity during ageing is often associated with peripheral chronic inflammation, and chronic neuroinflammation is a constant component of AD brain pathology. In the Special Issue published in 2021 eight papers were collected regarding different aspects of neurodegeneration associated with AD. Five papers presented and discussed infectious agents involved in brain AD pathology and three discussed data regarding receptors regulation and possible treatment of the disease. Below I will discuss and further elaborate on topics related to infections, inflammation, and neurodegenerative pathways in AD and brain senescence. The topic presented here may contribute to early intervention protocols for preventing or slowing the progression of cognitive deterioration in the elderly.

## 1. Alzheimer Disease, Cell Senescence, Chronic Inflammation, and Virus Infection

Alzheimer’s disease (AD) is a neurodegenerative disease and is responsible for most dementia cases in old age. Evidence indicates that abnormal protein accumulation in the brain is a relevant neurotoxicity mechanism observed in this form of dementia. However, brains of old people without cognitive impairment or dementia also show these amyloid deposits. Therefore, the role of these protein deposits in the etiology of the disease remains uncertain.

Cellular senescence is an emerging mechanism contributing to neurodegeneration in several diseases of central and peripheral nervous system. Under persistent stress, as ageing associated inflammation or chronic infections, neurons, and astroglia may enter cellular senescence (CS). This cellular state is characterized by division inactivation, resistance to apoptosis and production of pro-inflammatory molecules which in turn promote tissue functional decline. CS has been recently suggested as a non-secondary mechanism in neurodegeneration and dementia [1].

Pathogens, such as viruses of the Herpes family, through frequent cycles of reactivation and latency, constantly activate immune responses, which, however, cannot completely eradicate these infective agents. It has been suggested that these persistent neurotropic pathogens might play a role in microglia activation in the brain of genetically susceptible elderly and promote neurodegenerative processes [2].

On the other hand, amyloid-β (Aβ) peptide, associated with AD pathology, shows antimicrobial activity against microorganisms [3], and shares several characteristics with other antimicrobial peptides which are components of the innate immune system [3].

Our recent data showed that antimicrobial defense mechanisms of innate immunity appeared to be impaired in AD brains and we suggested that these immune alterations might contribute to dementia associated neurodegeneration [4].

Three articles from the 2021 Special Issue report data and discuss the role of viral infection in AD [5,6,7]. Mielcarska and coauthors describe changes in neurons, astrocytes, microglia, and oligodendrocytes related to the production of inflammatory factors, transition of glial cells into a reactive state, along with oxidative damage, Aβ secretion, tau hyperphosphorylation, apoptosis, and autophagy after infection by human herpes simplex virus-1 (HSV-1) [5].

I and E. Porcellini have discussed the role of exogenous infective viruses and endogenous retrovirus in human neurodegeneration and AD [6].

Chiricosta and coworkers have presented data regarding SAR-CoV-2 in human brain and concluded that SARSCoV-2 worsens the AD clinical condition by increasing neurotoxicity induced by higher levels of beta-amyloid, inflammation and oxidative stress in the AD brain [7].

New insights are emerging regarding CS induced by virus infection. For instance, latent infection by cytomegalovirus (CMV) induces immune senescence which in turn impairs immune responses to other pathogens, increases CS, and decreases vaccination efficacy [8]. Moreover, CMV infection promotes immune memory inflation which in turn increases the age associated mortality risk and accelerates unhealthy aging and CS [9].

Recurrent infection by HSV-1 has also been shown to significant increase neuronal aging in mice [10].

Another neurotropic virus, the human immunodeficiency virus (HIV) was shown to induce microglia senescence which can contribute to neurodegeneration [11] observed in this disease.

Recently, SARS-CoV-2 was shown to induce CS as a primary stress response of infected cells. This virus-induced senescence is indistinguishable from other forms of CS and is accompanied by the senescence-associated secretory phenotype (SASP) [12].

SARS-CoV-2 is indeed a neurotropic virus and can induce cognitive impairment and brain alterations during the acute infection clinical phase [6]. Whether this virus might induce a dysregulation of HERV in the brain and long-term alterations of brain metabolism remains an open question.

From the above data I conclude that some virus infections, even in persistence or latent forms, induce CS and accelerate tissue aging and brain neurodegeneration.

This notion is reinforced by other data showing that antiviral therapy in CMV infected mice reverses immune senescence during viral latent phase [13]. Therefore, infective viruses may contribute to neurodegenerative brain mechanisms by inducing CS.

Retroviruses have originated a small portion of the human genome. These ancient retroviruses, named human endogenous retroviruses (HERVs), were integrated into human genome [14]. Retrotransposon activity is responsible for the amplification of these integrated endogenous retrovirus (ERV) [15]. Some ERVs maintained some functions such as immune genes enhancers [16], and few also showed an infective capacity.

An abnormal activity of HERV has been described in human neurological diseases. For instance, levels of HERV-K have been found elevated in human amyotrophic lateral sclerosis [17]. On the other hand, increased HERV-W activation has been described in human multiple sclerosis [18].

Therefore, we recently suggested that brain ERV activation might represent a mechanistic and pathogenetic link of inflammation with brain aging and AD pathology [6].

Investigations regarding the role of retrotransposons and ERV in CS are scanty. However, recent results showed that mice injected with an HIV antigen (rVpr) increased the copy number of long interspersed element-1 in the heart genome. rVpr repeated injections also increased the number of cells positive for senescence-associated β-galactosidase and induced heart fibrosis [19]. In conclusion, I hypothesize that, abnormal brain activation of HERV, by inducing CS of neuronal cells, might be an additional mechanism of neurodegeneration.

The article from Van Thi Ai and collaborators [20] in the Special Issue described that bacteria, usually infecting the oral cavity, induces microgliosis and neurodegeneration in an in vitro model of neural cell platform. They conclude that the mouth-brain axis may contribute to the pathogenesis of AD. Therefore, I may infer that also bacterial infection may cause CS. Evidence shows that CS is indeed induced by pathogens, and can be mediated directly through virulence determinants or indirectly through inflammation and chronic infection [21].

The article from Tawfic and collaborators [22] published in the Special Issue shows that hyperhomocysteinemia (HHcy) is common among the elderly. The relation between HHcy and the development of neurodegenerative diseases, such as Alzheimer’s disease (AD), the age-related macular degeneration (AMD) and diabetic retinopathy (DR) in old people has been discussed in their paper. It is of interest that HHcy increases inflammatory responses and therefore, it may induce CS. HHcy was indeed recently reported to induce neuronal senescence [23].

Investigation from Alabed and coworkers [24] published in the Special Issue showed that methamphetamine (METH) impacts AD by modulating amyloid precursor protein (APP) expression. However, they showed that METH also increases the production of inflammatory mediators, and mediates the disruption of the blood–brain barrier. Therefore, METH by activating brain inflammatory response may also be an inducer of CS. In fact, METH has been recently shown to cause CS in pulmonary cells [25].

Finally, another paper in the Special Issue by Grossman [26] suggests a treatment with a thrombin inhibitor to modulate AD brain inflammation. I suggest that a possible effect of this drug consists of decreasing inflammatory responses induced by HERV or exogenous pathogens and modulating neurodegenerative process associated with dementia.

**Figure 1:**
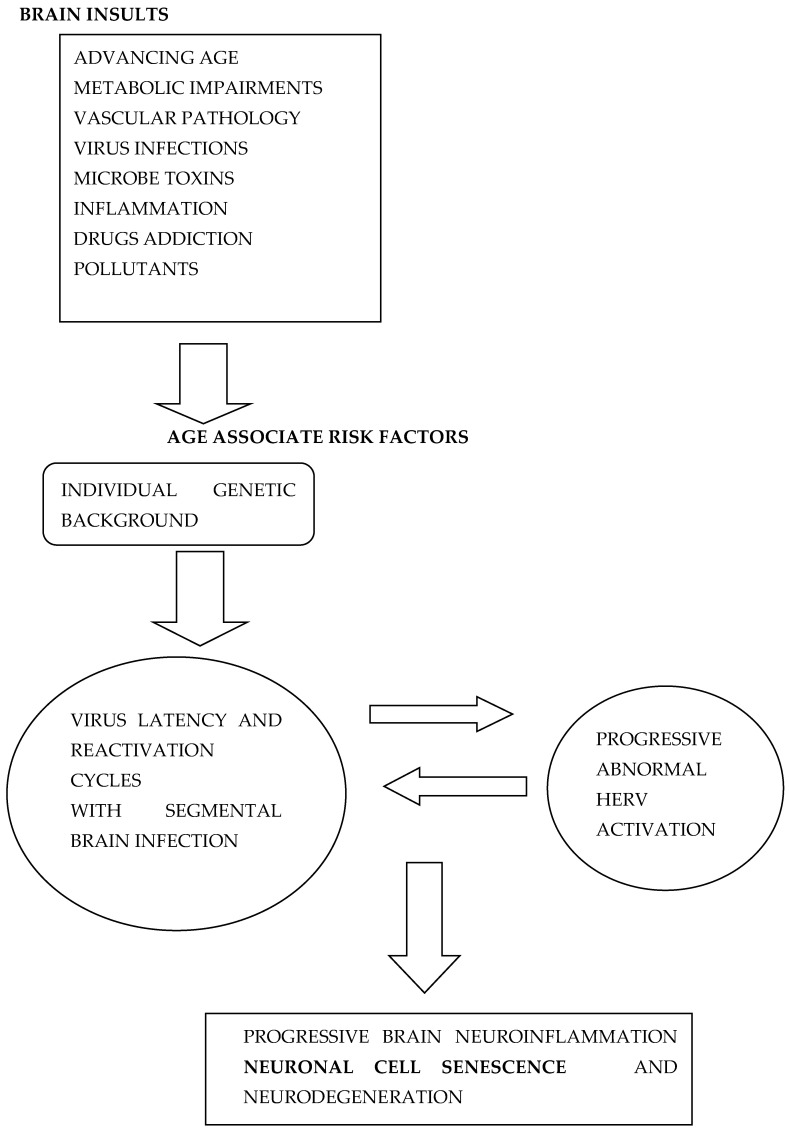
Different age-related risk factors contribute to the interplay between exogenous virus infections and HERV abnormal activation leading to neuronal cell senescence and further neurodegenerative processes and AD.

Several different mechanisms appear to induce brain inflammation and neuronal senescence (Figure 1) which in turn may contribute to neurodegeneration associated with AD. Other factors such as age, gender genetic makeup, smoke, pollutants, and diet also contribute to neurodegenerative processes. Here, I discuss that CS might be a non-secondary mechanism associated with AD and a convergence’s point induced by different damaging processes in human brain which amplify degenerative processes. Moreover, several natural or synthetic compounds have shown anti-CS activity [27] and have been called senolytic drugs. Some of these drugs might be used for prevention of cognitive decline and dementia in the near future.

## 2. Conclusions

Notions discussed in 2021 Special Issue “Infections, Inflammation and Neurodegeneration in Alzheimer Disease” have relevant implications for the prevention and treatment of prodromal AD and its early clinical phase. I have been pleased to further discuss the topic in this Editorial and present the Special Issue’s contribution to the AD field, by elaborating on a possible link between chronic inflammation and CS.
